# Flavanones from *Erythrina crista-galli* Twigs and Their Antioxidant Properties Determined through In Silico and In Vitro Studies

**DOI:** 10.3390/molecules27186018

**Published:** 2022-09-15

**Authors:** Vanny Deviani, Ari Hardianto, Kindi Farabi, Tati Herlina

**Affiliations:** Department of Chemistry, Faculty of Mathematics and Natural Sciences, Universitas Padjadjaran, Jatinangor 45363, West Java, Indonesia

**Keywords:** *Erythrina crista-galli*, flavanone, antioxidant, DPPH, DFT, donor acceptor map

## Abstract

Flavonoids are a secondary metabolite group with various bioactivities, such as antioxidants. They are rich in the genus *Erythrina*, such as *Erythrina crista-galli*. This research aims to isolate and characterize flavonoids from the twigs of *E. crista-galli* and determine their antioxidant properties through in silico and in vitro assays. The ethyl acetate extract of *E. crista-galli* twigs were separated by column chromatography and characterized using spectroscopic methods. Density functional theory (DFT) calculations were performed on the isolated flavonoids and the reference compounds (ascorbic acid and quercetin) to obtain global descriptive parameters and a donor–acceptor map (DAM). We successfully isolated lupinifolin (**1**) and citflavanone (**2**) for the first time from *E. crista-galli*, along with lonchocarpol A (**3**), which has been discovered previously. The DAM suggests that these flavanones are good antiradicals with effective electron donors. However, they tend to be electron acceptors in methanol. The frontier molecular orbital analysis implies that lupinifolin (**1**) is a better antiradical than the other flavanones. The DPPH assays show that lupinifolin (**1**) has the highest antioxidant (antiradical) activity, with an IC_50_ value of 128.64 ppm. The in silico studies showed similar trends to the in vitro assays using the DPPH method.

## 1. Introduction

*Erythrina* is one of the plants rich in flavonoids [1]. A total of 409 flavonoids have been reported in this genus and are grouped according to their chemical structures [2]. The genus *Erythrina* (family Fabaceae) has 290 species, distributed in tropical and subtropical regions around the world, and only 130 of them have been studied [3]. They are spread all over Indonesia, from Sumatra to Papua. Many Indonesians use them as traditional medicines to treat various diseases, such as malaria, cough, asthma, and microbial infections [4,5].

*E. crista-galli* is a member of the genus *Erythrina*, which can be found in Indonesia, Australia, Argentina, Uruguay, Paraguay, and Brazil. In Indonesia, it is known as “dadap merah” [6]. Since various flavonoids are discovered in the genus *Erythrina*, *E. crista-galli* may also contain a wide variety of these metabolites. However, the exploration of flavonoids in this species is still limited, with only 18 flavonoids reported so far [1]. 

Flavonoids have various bioactivities, such as antioxidants, which are critical for health [1]. The importance of antioxidants today is in line with the high air pollution, which indeed contains free radicals that can damage essential biomolecules in cells. The average level of free radicals in cells can be overcome by internal antioxidants produced in the body. Nevertheless, a higher number of free radicals than internal antioxidants causes the formation of oxidative stress. Oxidative stress can lead to several diseases, such as cancer, heart disease, cataracts, premature aging, neurological diseases, ischemia/perfusion, diabetes, asthma, and other degenerative disorders [7]. Therefore, additional antioxidants from outside the body are needed. Flavonoids as supplemental antioxidants are essential to help scavenge free radicals that internal antioxidants cannot overcome.

Flavanones are a class of flavonoids with antioxidant activities due to their phenolic moieties [1]. They may follow an electron transfer mechanism in scavenging free radicals [8]. This mechanism involves the donating or accepting of electrons; thus, hereafter, we refer to antioxidants as antiradicals [9,10,11]. The mechanism of electron transfers is as shown below:

*aox* + *R·* → *aox*(+) + *R*(−)

where *aox* represents an antiradical, *R·* represents a free radical, *aox(+)* represents a radical form of antiradical, and *R*(−) represents the neutralized free radical, assuming that antioxidants must lose or donate electrons to neutralize free radicals. The electron transfer mechanism analysis requires a computation of the ionization potential (*I*) parameter. A low *I* value indicates good antiradical activity, since it reflects the ease of an electron abstracted from an antiradical molecule. Electron affinity (*A*) is another essential parameter in electron transfer mechanism analysis because antiradicals can act as either electron donors or acceptors, which means that the following reactions may occur:

*aox* + *R·* → *aox*(−) + *R*(+)

**Figure 1:**
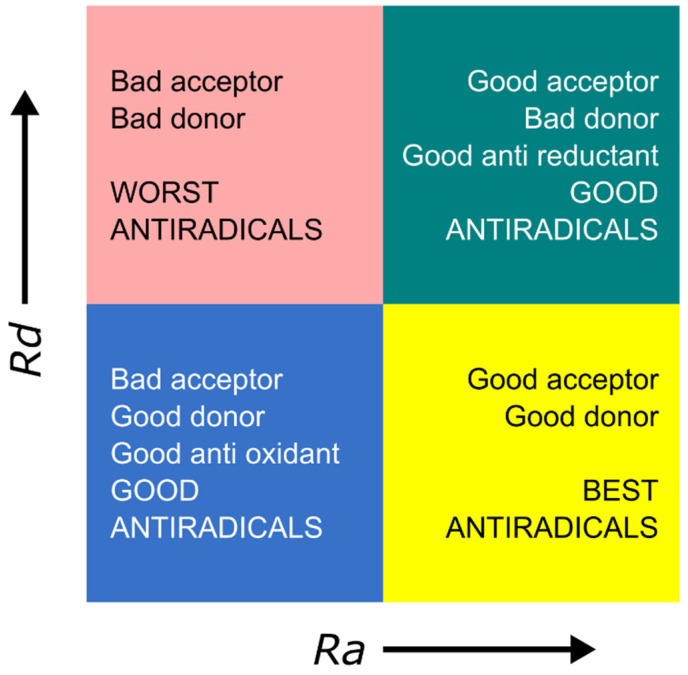
Donor–acceptor map (DAM).

Therefore, computations of *I* and *A* are essential in analyzing the antiradical ability of a compound. These parameters are obtained using quantum chemical calculations on active compounds of interest and known antioxidants (antiradicals), such as ascorbic acid and quercetin. Moreover, the electron-donating (*Rd*) and electron-accepting indices (*Ra*) are calculated for sodium and fluorine atoms, respectively, as references. Next, *Ra* and *Rd* values are plotted to construct the donor–acceptor map (DAM) [9]. The DAM (Figure 1) is helpful as a qualitative comparison tool between compounds, by classifying molecules based on their electron-accepting and electron-donating abilities. The DAM can be seen as a good illustration in helping to reveal the antiradical capacity of any substance because the electron transfer reaction represents one of the methods used for free radical scavenging [9,12].

Through this paper, we report the isolation of flavanones in *E. crista-galli* twigs and determine their antioxidant activity by in silico and in vitro assays.

## 2. Results and Discussion

### 2.1. Extraction and Isolation

The methanol extract of *E. crista-galli* twigs was dissolved in water and partitioned with *n*-hexane and ethyl acetate. Subsequently, we selected the ethyl acetate fraction for further purification steps, due to the similar polarity between flavonoids and the solvent [13,14,15]. The flavonoid phytochemical test using NaOH gave a positive result, which supported our decision. The ethyl acetate extract was then separated by column chromatography, either through the normal phase or the reversed phase, to yield three isolates. Their structures were identified by UV, HR-TOFMS, IR, 1D, and 2D NMR.

**Figure 2:**
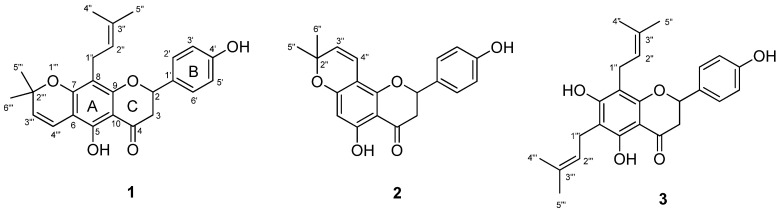
Chemical structures of flavanones isolated from *E. crista-galli.*

Lupinifolin (**1**) (Figure 2) was obtained as a yellow oil, which appeared as a dark purple spot on the TLC under UV light and produced a yellow stain after spraying with AlCl_3_. UV (MeOH) *λ_max_* nm: 313 and 275; +NaOH 380 and 291. IR (KBr Pellets) *ν_max_* cm^−1^: 3367, 2974, 2925, 1626, 1449, 1120, 834. HR-TOFMS m/z 407.1859 [M+H]^+^ (calcd. for C_25_H_27_O_5_^+^ 407.1858). ^1^H-NMR (500 MHz, CDCl_3_): *δ_H_* 12.23 (s, 5-OH); 7.31 (d, *J* = 8.5 Hz, H-2′; H-6′); 6.89 (d, *J* = 8.5 Hz, H-3′, H-5′); 6.62 (d, *J* = 10 Hz, H-4‴); 5.49 (d, *J* = 10 Hz, H-3‴); 5.36 (br s, 4′-OH); 5.32 (dd, *J* = 3.5; 13.5 Hz, H-2); 5.12 (t, *J* = 7.5 Hz, H-2″); 3.19 (d, *J* = 7.5 Hz, H-1″); 3.03 (dd, *J* = 13.5; 17 Hz, H-3ax); 2.80 (dd, *J* = 3.5; 17 Hz, H-3eq); 1.63 (s, H-4″; H-5″) and 1.41 ppm (s, H-5‴; H-6‴). ^13^C-NMR (125 MHz, CDCl_3_): *δ_C_* 196.5 (C-4), 159.9 (C-7), 159.4 (C-9), 156.6 (C-5), 155.9 (C-4′), 131.2 (C-1′), 131.0 (C-3″), 127.8 (C-2′, C-6′), 126.0 (C-3‴), 122.5 (C-2″), 115.7 (C-4‴), 115.6 (C-3′, C-5′), 108.7 (C-8), 102.8 (C-6), 102.7 (C-10), 78.6 (C-2), 78.2 (C-2‴), 43.3 (C-3), 28.4 (C-6‴), 28.3 (C-5‴), 25.9 (C-4″), 21.5(C-1″), and 17.9 (C-5″) ppm. As a result, the chemical shift of compound **1** was similar to lupinifolin. Thus, compound **1** was identified as lupinifolin, which was previously isolated from the stem bark of *E. fusca* [16]. Nevertheless, this was the first time it was isolated from *E. crista-galli*.

Citflavanone (**2**) (Figure 2) was obtained as a yellow oil, which appeared as a dark purple spot on the TLC under UV light and produced a yellow stain after spraying with AlCl_3_. UV (MeOH) *λ_max_* nm: 305 and 272; +NaOH 345 and 287. IR (KBr Pellets) *ν_max_* cm^−1^: 3357, 2973, 2926, 1633, 1448, 1155, 834. HR-TOFMS m/z 339.1239 [M+H]^+^ (calcd. for C_20_H_19_O_5_^+^ 339.1232). ^1^H-NMR (500 MHz, CDCl_3_): *δ_H_* 12.28 (s, 5-OH); 7.30 (d, *J* = 8.5 Hz, H-2′; H-6′); 6.87 (d, *J* = 8.5 Hz, H-3′, H-5′); 6.59 (d, *J* = 9.5 Hz, H-4″); 5.93 (s, H-6); 5.48 (d, *J* = 9.5 Hz, H-3″); 5.31 (dd, *J* = 3; 13 Hz, H-2); 3.06 (dd, *J* = 13; 17 Hz, H-3ax); 2.77 (dd, *J* = 3; 17 Hz, H-3eq) and 1.42 ppm (s, H-4″; H-5″). ^13^C-NMR (125 MHz, CDCl_3_): *δ_C_* 196.1 (C-4), 162.4 (C-7), 162.2 (C-9), 158.4 (C-5), 156.2 (C-4′), 130.5 (C-1′), 128.0 (C-2′, C-6′), 126.3 (C-3″), 115.7 (C-3′, C-5′), 115.3 (C-4″), 103.1 (C-10), 102.9 (C-8), 96.3 (C-6), 78.9 (C-2), 78.4 (C-2″), 43.2 (C-3), 28.3 (C-5″), and 28.2 (C-6″) ppm. To strengthen the alleged structure of compound **2**, a study of the literature was conducted by comparing spectroscopic data of ^1^H-NMR and ^13^C-NMR. As a result, the chemical shift of compound **2** was similar to citflavanone. Thus, compound **2** was identified as citflavanone, which was firstly discovered in *E. crista-galli*. Like compound **1**, its isolation from the stem bark of *E. fusca* was previously reported [16].

Lonchocarpol A (**3**) (Figure 2) was obtained as a yellow oil, which appeared as a dark purple spot on the TLC under UV light and produced a yellow stain after spraying with AlCl_3_. UV (MeOH) *λ_max_* nm: 326 and 277; +NaOH 382 and 312. IR (KBr Pellets) *ν_max_* cm^−1^: 3372, 2965, 2920, 2855, 1632, 1446, 835. ^1^H-NMR (500 MHz, CDCl_3_): *δ_H_* 12.31 (s, 5-OH), 7.30 (d, *J* = 8.5 Hz, H-2′; H-6′), 6.87 (d, *J* = 8.5 Hz, H-3′, H-5′), 5.31 (dd, *J* = 3.5;13 Hz, H-2); 5.21 (t, *J* = 7 Hz, H-2″); 5.17 (t, *J* = 7 Hz, H-2‴); 3.32 (d, *J* = 7 Hz, H-1‴); 3.27 (d, *J* = 7 Hz, H-1″); 3.02 (dd, *J* = 13; 17 Hz, H-3ax); 2.78 (dd, *J* = 3.5; 17 Hz, H-3eq); 1.80 (s, H-4″); 1.73 (s, H-5″); 1.69 (s, H-5‴) and 1.68 ppm (s, H-4‴). ^13^C-NMR (125 MHz, CDCl_3_): *δ_C_* 196.6 (C-4), 162.4 (C-7), 159.3 (C-5), 157.8 (C-9), 155.9 (C-4′), 134.8 (C-3″), 134.1 (C-3‴), 131.1 (C-1′), 127.7 (C-2′, C-6′), 122.0 (C-2‴), 121.8 (C-2″), 115.5 (C-3′, C-5′), 107.3 (C-6), 106.5 (C-8), 102.8 (C-10), 78.5 (C-2), 43.3 (C-3), 25.9 (C-5″, C-5‴), 21.9 (C-1″), 21.3 (C-1‴), and 17.9 (C-4″, C-4‴) ppm. Compound **3** was identified as lonchocarpol A, which was previously reported to be found in the stem bark of *E. crista-galli* [5].

The structures of lupinifolin (**1**), citflavanone (**2**), and lonchocarpol A (**3**) are depicted in Figure 2. 

### 2.2. Antioxidant Properties through in Silico Studies

#### 2.2.1. The Global Descriptive Parameters 

We employed the global descriptive parameters to analyze the reactivity of flavanones and other reference molecules quantitatively. In light of this, we computed ionization potential and electron affinity values for all molecules using a single point energy method [10]. Table 1 reports the values of the global descriptive parameters of the isolated flavanones and reference compounds, quercetin and ascorbic acid. These parameters depend on the number of electrons and the electron density. The global descriptive parameters included electron affinity (*A*), ionization potential (*I*), hardness (*η*), softness (*S*), electronegativity (*χ*), chemical potential (*μ*), and electrophilicity index (*ω*).

The stability of molecules was directly associated with the hardness parameter (*η*), whereas softness (*S*) provided information about the chemical reactivity of molecules [17]. Flavanones have higher chemical hardness (*η*) values than standard compounds in the gas phase, due to their higher stabilities. The parameter’s order of softness was quercetin > ascorbic acid > lupinifolin (**1**) > citflavanone (**2**) > lonchocarpol A (**3**), respectively, indicating that standard compounds were more favorable for the charge–transfer mechanism than all flavanones. However, lupinifolin (**1**) had a better chance of transferring charge than the others among all three flavanones. 

According to Table 1, all flavanones had lower electronegativity (*χ*) values than the standard compounds, suggesting their electron-donating properties and greater antiradical capacity. Meanwhile, the chemical potential (*μ*) values of flavanones (Table 1) were more positive than those of the standard compounds. These data indicate that flavanones tended to lose an electron: the more negative the chemical potential is, the easier it is for the molecule to accept an electron [17].

#### 2.2.2. Ionization Potential and Electron Affinity

The calculated electron affinities (*A*) and ionization potentials (*I*) for all compounds studied here are tabulated in Table 1, where quercetin and ascorbic acid are used as comparisons. The isolated flavanones (**1**–**3**) had lower *I* values than quercetin and ascorbic acid. According to their ability to donate electrons, compounds with low *I* values are readily oxidized and are hence good antiradicals [9]. Therefore, flavanones (**1**–**3**), especially lupinifolin (**1**), may act as more potent antioxidants than quercetin and ascorbic acid. 

Regarding electron affinity (*A*), quercetin and ascorbic acid both had positive and larger values, whereas flavanones had negative or low positive values (lupinifolin). Thus, flavanones were less effective electron acceptors than quercetin and ascorbic acid, in terms of their electron-accepting capacity.

Substances must either give or take electrons to scavenge free radicals. Based on their ability to donate electrons, we may conclude that flavanones, particularly lupinifolin (**1**), serve as better antiradicals compared to ascorbic acid and quercetin.

#### 2.2.3. Electro-donating (*ω*^−^) and Electro-accepting Power (*ω*^+^)

Low electro-donating power values (*ω*^−^) are necessary for effective electron donors, while high electro-accepting power (*ω^+^*) values denote efficient electron acceptors [9]. Based on Table 2, we show that flavanones have a greater electro-donating power than ascorbic acid and quercetin. According to the *ω*^−^ value, the order of reactivity expressed in terms of oxidation facility is as follows:

Flavanones > quercetin > ascorbic acid

where flavanones may be good antioxidants which prevent the oxidization of free radicals by donating their electrons, whereas ascorbic acid and quercetin are bad ones. 

Analyzing the second process for electron transfer, known as electron capture, was crucial to figuring out the antiradical level of a substance. Below is the order of reactivity based on the values of *ω^+^*:

Ascorbic acid > quercetin > flavanones

where ascorbic acid and quercetin represent the good anti-reductant, whereas flavanones represent the worst. 

Both *ω*^−^ and *ω^+^* values (Table 2) suggest that flavanones or standard compounds are antiradical, either antioxidant or anti-reductant. It is worth noting that flavanones and standard compounds use different scavenging mechanisms to quench free radicals. Flavanones are good electron donors, whereas ascorbic acid and quercetin are good electron acceptors.

#### 2.2.4. Donor Acceptor Map (DAM)

**Figure 3:**
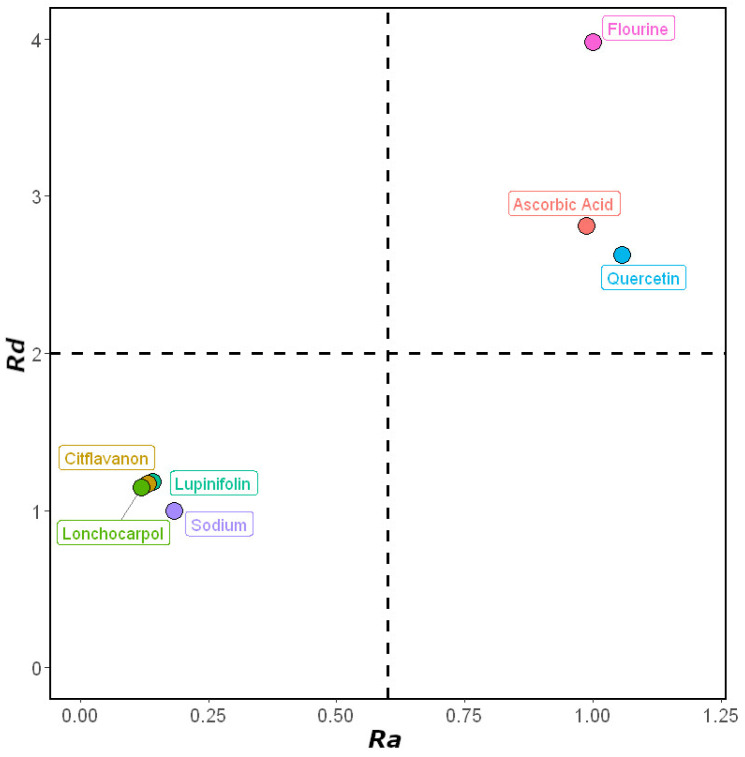
DAM of flavanones (**1–3**) and reference compounds (Table S1).

*Ra* and *Rd* values for flavanones **1–3**, accompanied by quercetin and ascorbic acid as standard compounds, are shown in Table 2 and visualized as a DAM (Figure 3). The DAM is useful as a qualitative comparison tool between compounds by classifying molecules based on their ability to accept and donate electrons [12]. It was successfully created through computational calculations using the DFT/B3LYP/6-311+G(2d,2p) method. Based on the DAM plot (Figure 3), flavanones (**1–3**) are compounds that are classified as good anti-radical compounds (good antioxidant sector). Ascorbic acid and quercetin are located in a good antiradical zone with good anti-reductant characteristics. 

When flavanones were compared with ascorbic acid, the former was found to be a better antioxidant; conversely, ascorbic acid and quercetin represented greater anti-reductants than flavanones. Ascorbic acid and quercetin have the potential to act as both an anti-reductant and an antiradical. Flavanones, on the other hand, are able to scavenge free radicals more effectively than standard compounds, mainly by donating electrons, but their ability to receive electrons is quite poor. However, as shown in Figure 3, the electron donor properties of flavanones are weaker than that of sodium.

These findings cannot be used to claim that flavanones are better at scavenging free radicals than standard compounds, or vice versa, because the processes used to do so are different. As antiradical agents, flavanones are better antioxidants, whereas ascorbic acid and quercetin are better anti-reductants. Ascorbic acid or quercetin may be more efficient against oxidative stress than flavanones in some situations. However, with a different free radical, or under different conditions, flavanones may perform better as an antiradical. Additionally, a living organism’s chemical environment, the solubility of molecules in various solvents, and the location of these antiradical molecules’ sites of action are all significant factors in scavenging free radicals [9].

#### 2.2.5. Solvent Effect

**Figure 4:**
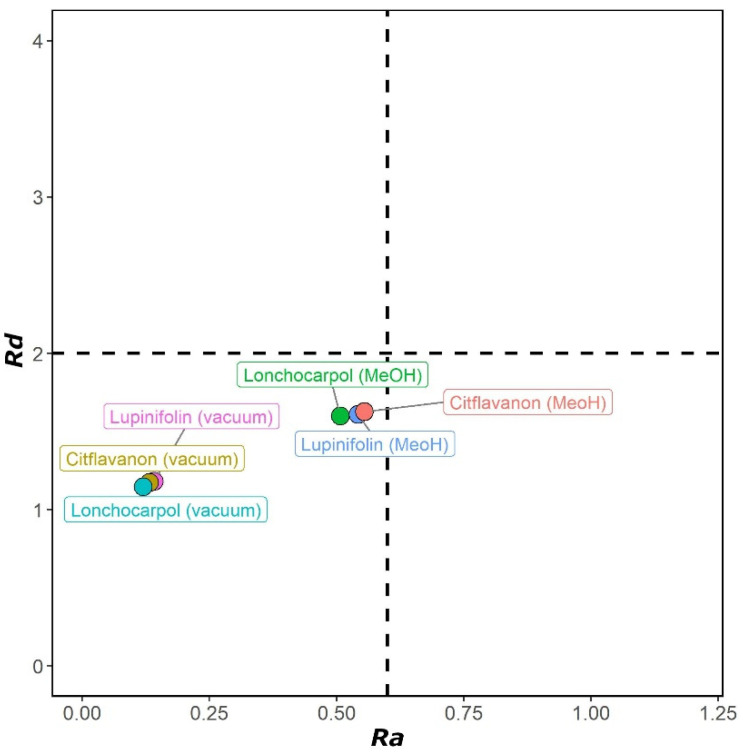
DAM for flavanones (**1–3**) in the gas phase and implicit methanol solvent (Table S1).

*I* and *A* values for all flavanones were calculated using the solvent effect (methanol). The results are presented in Table 3 and visualized in Figure 4. The relative order was the same when utilizing solvents and during the gas phase. In comparison to those in the gas phase, the implicit methanol solvent caused *I* values to decrease, while *A* values increased, with the same overall pattern. However, as shown in Figure 4, the solvent significantly impacted *Ra* and *Rd* values. The three flavanones in methanol were less efficient electron donors but were better electron acceptors than during the gas phase. 

#### 2.2.6. Frontier Molecule Orbital analysis

**Figure 5:**
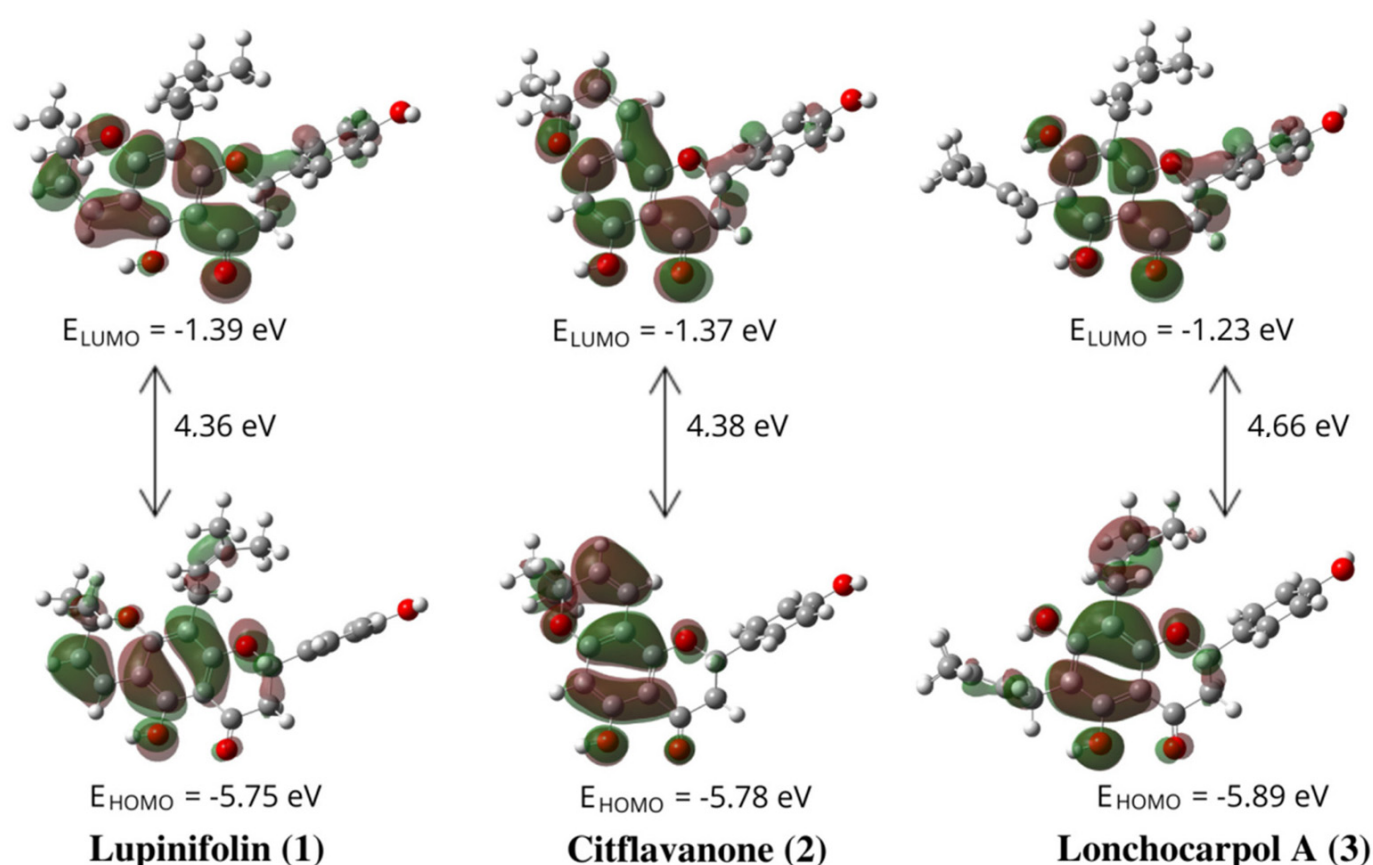
The energy and distribution of frontier orbital (HOMO and LUMO) of flavanones (**1–3**) in the gas phase.

Another important factor that correlates with the antiradical action is the energy and distribution of frontier orbitals [18]. Figure 5 shows the gas-phase electron-density distribution, the energy of the highest occupied molecular orbital (HOMO), and the lowest unoccupied molecular orbital (LUMO) for flavanones (**1–3**)**.**

The energy of the HOMO is a significant chemical characteristic connected with the free radical scavenging potential. Molecules with more positive HOMO energy values have a higher ability to donate electrons. Additionally, by analyzing the electronic density distribution in these orbitals, it was possible to predict the most likely sites of flavanones where free radicals will attack the compounds most readily [18]. Figure 5 shows that lupinifolin (**1**) had the highest HOMO energy (−5.7590 eV), followed by citflavanone (**2**) (−5.7835 eV), and lonchocarpol A (**3**) (−5.8950 eV). These results suggest that lupinifolin (**1**) has the most potential to donate electrons among other flavanones. In addition, the order of predicted electron-donating abilities based on the HOMO energy was the same as the *I* values (Table 1). Figure 5 shows that the LUMO and HOMO compositions exhibit similar distributions for the three flavanones. The electronic density of the HOMO and LUMO was mainly localized on the A-ring of the flavanone structure, 2,2-dimethylpyran, prenyl, and carbonyl groups, thus indicating that they were the most likely sites for radical attack. The presence of the prenyl and 2,2-dimethylpyran groups in lupinifolin (**1**) makes improved the antiradical activity of this compound, compared to the other two flavanones, which were supported based on electron density and higher HOMO energy, while having the smallest energy gap.

### 2.3. DPPH Radical Scavenging Assays 

All extract fractions of *E. crista-galli* twigs were tested for their antioxidant activity against DPPH (Table 4). Another consideration in selecting ethyl acetate extract was regarding its strong antioxidant activity against DPPH radicals, with an IC_50_ value of 64.41 ppm. This antioxidant activity was expected to have a synergistic effect with the pure compounds obtained later. Flavanones (**1**–**3**), isolated from the ethyl acetate extract of *E. crista-galli* twigs, were tested for their antioxidant activity against DPPH radicals so that the IC_50_ value of each compound was compared with quercetin and ascorbic acid as positive controls (Table 4). Based on the experimental results, lupinifolin (**1**) had a lower IC_50_ value than citflavanone (**2**) and lonchocarpol A (**3**). The lower IC_50_ value indicates that the antioxidant activity was better. Lupinifolin (**1**) had moderate antioxidant activity when compared to the standard compounds. The presence of a prenyl group at the C-8 position, and the presence of a pyran group in lupinifolin (**1**), are thought to be interesting compared to citflavanone (**2**) and lonchocarpol A (**3**). The electron-donating group in flavanone compounds can encourage ring activation in flavanones, and the presence of pyran increases the length of the conjugated double bond, which can induce an electron shift through a resonance effect to form more stable flavanone radicals and increase antioxidant activity [19].

**Figure 6:**
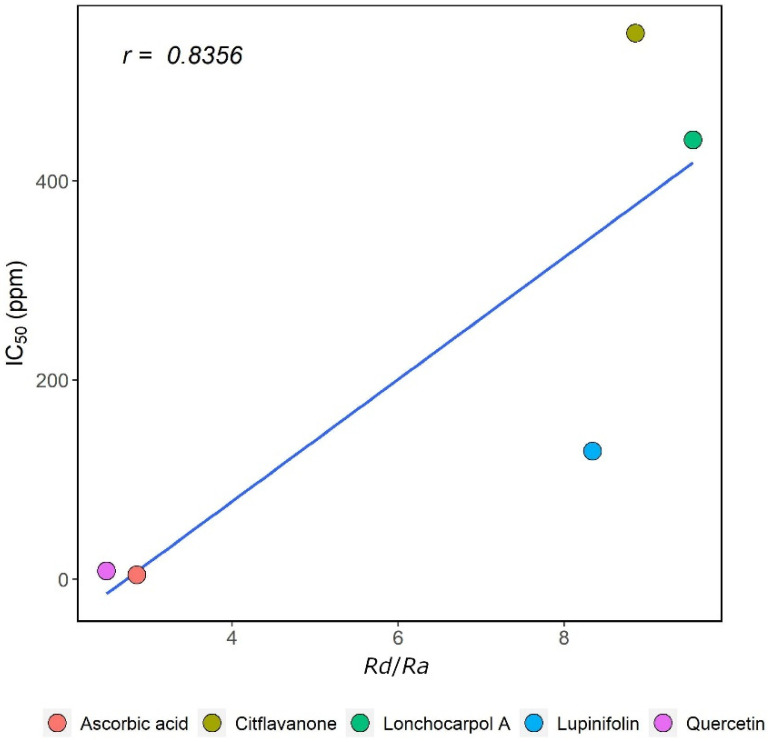
Pearson correlation coefficient between the results of in silico and in vitro studies (Table S1).

A correlation coefficient calculation is needed to determine the relationship between in silico and in vitro studies. A correlation coefficient is data in the form of a value that shows the size of a linear and logical relationship between variables X and Y [20]. Figure 6 shows the Pearson correlation coefficient (*r*) between the results of in silico and in vitro studies for flavanones (**1–3**) and standard compounds. The value of *r* was 0.8356, which indicates that the correlation between in silico and in vitro studies had a good correlation. However, lupinifolin (**1**) and citflavanone (**2**) deviated from the regression line with residuals of −215.65 and 172.81 ppm, respectively. Overall, both studies showed a similar trend in antiradical activities based on their correlation coefficient. 

## 3. Materials and Methods

### 3.1. General

The UV spectrum was measured with a Thermo Scientific G10S UV-Vis (Thermo Fisher Scientific, Madison, WI, USA). Infrared (IR) absorption measurements were carried out with a KBr plate using a One Perkin Elmer spectrum-100 FTIR spectrometer (Perkin Elmer, Beaconsfield, United Kingdom). The NMR spectra were recorded with a 500 MHz JEOL Delta NMR spectrometer (^1^H-NMR, ^13^C-NMR, and 2D NMR) (Jeol, JNM ECA 500, Tokyo, Japan). Determination of relative molecular mass was conducted using a high-resolution of mass spectra (HR-TOFMS) on a Waters Xevo Q-TOF direct probe/MS system, utilizing ESI mode and a microchannel plate MCPs detector (Thermo Fisher Scientific, Milford, MA, USA). The separation was guided using thin layer chromatography (TLC) with a Vilbert Luomart UV detector lamp (254 and 365 nm). Chromatography was performed using octa desylsilane (Chromatorex® C18 DM1020 M, 200–400 mesh, Fuji Sylisia, Tokyo, Japan) and silica gel 60 (70–230 and 230–400 mesh, Merck, Darmstadt, Germany). Silica gel GF254 (0.25 mm, Merck, Darmstadt, Germany) was used for thin layer chromatography (TLC). The spot detection on TLC was visualized under UV light and sprayed with stain-seeking reagent 10% H_2_SO_4_ in ethanol and AlCl_3_ in ethanol, followed by heating.

### 3.2. Plant Material

Samples of *E. crista-galli* twigs were obtained from Jl. Sersan Bajuri, Bandung, West Java, Indonesia. This plant was previously identified in the Laboratory of Agricultural Production Technology and Services, Department of Agricultural Cultivation, Faculty of Agriculture, Universitas Padjadjaran, and the voucher specimen number 1020 was stored in the Herbarium. Twigs were cleaned, dried in the open air, cut into small pieces, and ground. The dried sample was stored in a dry room under room-temperature conditions.

### 3.3. Extraction and Isolation

*E. crista-galli* twig powder (4 kg) was extracted with methanol using a maceration technique and concentrated with a rotatory evaporator to obtain a concentrated methanol extract (115 g). The concentrated methanol extract was partitioned with *n*-hexane and ethyl acetate to obtain *n*-hexane (20.8 g) and ethyl acetate (34.6 g) extracts. The ethyl acetate extract (25 g) was fractionated by the vacuum liquid chromatography (KCV) method using *n*-hexane:ethyl acetate:methanol as an eluent in a 10% gradient to produce nine combined fractions (A-I), based on the TLC results. Fraction D (901 mg) was separated by silica gel column chromatography using *n*-hexane:ethyl acetate:methanol gradient 5% to produce 12 subfractions (D1-D12). Subfractions D9 (140 mg) and D10 (110 mg) were purified by column chromatography and eluted with a mixture of *n*-hexane:chloroform:ethyl acetate (7:2:1) to produce lupinifolin (**1**) (19.8 mg, 0.0005% yield). The D11 subfraction was separated by column chromatography of silica gel and the eluent *n*-hexane:ethyl acetate (8:2) to produce 5 subfractions (D11A-D11E). The D11D subfraction was separated by ODS column chromatography, which was eluted with methanol:water (9:1) to produce three subfractions (D11d1–D11d3). Subfraction D11d2 was purified by silica gel column chromatography with gradient elution of *n*-hexane:acetone, 1% yielded citflavanone (**2**) (13.3 mg, 0.0003% yield) and lonchocarpol A (**3**) (6.5 mg, 0.0002% yield).

### 3.4. Computational Methods

An in silico study was devoted to predicting the antioxidant activity of isolated flavanone compounds and was calculated based on the method described in the literature [9,12] with several modifications. Calculations were carried out using Gaussian 09 software (Gaussian, Wallingford, CT, USA). The structures of the isolated and standard compounds were downloaded from PubChem (https://pubchem.ncbi.nlm.nih.gov/; accessed on 2 December 2021). The three-dimensional structure of the compound was analyzed using Marvin Suite 18.21.0 (ChemAxon, Sydney, NSW, Australia) to obtain a suitable structure at pH 7.4. Geometry optimization (default setting) was carried out using the B3LYP function DFT method with a basis set of 6-31+G(2d,2p). The structure of the optimization results was confirmed by frequency analysis using the same method (B3LYP/6-31+G(2d,2p)) to ensure that the minimum value (without imaginary frequency) was obtained. The unrestricted open-shell approach was also used for radical species. No spin contamination was found, and the *S*^2^ values were around 0.750 for all radical species. The frontier molecular orbitals (FMOs), in particular, are made up of the highest occupied molecular orbital (HOMO) and the lowest unoccupied molecular orbital (LUMO). Density functional theory was used to analyze the frontier molecular orbitals of substance and their energy gaps were calculated. A lower energy gap indicated the reactivity of the molecule.

Global descriptive parameters provided the reactivity of compounds. The global descriptive parameters included the electron affinity (*A*), ionization potential (*I*), hardness (*η*), softness (*S*), electronegativity (*χ*), chemical potential (*μ*), and electrophilicity index (*ω*). Based on the vertical energy, the difference in the total electronic energy of the neutral molecule and the corresponding anions and cations was considered. Solvent effects were included, using the default model (polarizable continuum model) of Gaussian with a methanol solvent. The following equations can be used to determine electron affinity (*A*) and ionization potential (*I*) [10,12];

*I* = E_cation_ − E_neutral_ (1)

*A* = E_neutral_ − E_anion_ (2)

The formulae listed below were used to calculate the global properties [12,17]:

*η* = (*I* − *A*)/2 (3)

*S* = 1/(2*η*) (4)

*χ* = (*I* + *A*)/2 (5)

*μ* = −*χ* (6)

*ω* = *μ*^2^/2 (7)

The tendency of charge donation or electron-donating power is as follows [10,12]:

*ω*^−^ = (3*I* + *A*)^2^/16 (*I* − *A*) (8)

whereas, the propensity to accept charge or electron-accepting power is expressed as [10,12]:

*ω^+^* = (*I* + 3*A*)^2^/16 (*I* − *A*) (9)

A higher capacity for donating charge is implied by lower electron-donating power values. Higher electro-accepting power values indicate a larger capacity to accept charge. 

The electron-accepting indices (*Ra*) for all compounds (*C*) were calculated using the equation below [10]:

(10) $$Ra=\frac{\omega_{C}^{+}}{\omega_{F}^{+}}$$

where $\omega_{C}^{+}$ and $\omega_{F}^{+}$ are electron-accepting power values for the compound of interest and the fluorine atom, respectively. The $\omega_{F}^{+}$ was derived from the experimental values of *A* and *I* for the fluorine atom [21,22], using Equation (9). The fluorine atom was used since it is a good electron acceptor [9].

The electron-donating index (*Rd*) for all test samples (*C*) was calculated as [10]:

(11) $$d=\frac{\omega_{C}^{-}}{\omega_{Na}^{-}}$$

where $\omega_{C}^{-}$ and $\omega_{Na}^{-}$ are electron-donating power values for the compound of interest and the sodium atom, respectively. The sodium atom was used due to its good electron-donor property [9]. We computed $\omega_{Na}^{-}$ by subjecting the values of *A* and *I* for the sodium atom, obtained from the literature [23,24], to Equation (8). 

After both *Ra* and *Rd* values were calculated for all compounds, we constructed the donor acceptor map (DAM).

### 3.5. DPPH Radical Sscavenging Assays

The free radical scavenging activity was evaluated based on the method described in the literature [25] with some modifications. The test sample was mixed with 60 μL of DPPH solution in 4.10^–4^ M methanol. The mixture was then incubated for 30 min in a dark room. The absorbance was measured using a microplate reader at a maximum wavelength of 510 nm. Ascorbic acid and quercetin were used as reference standards.

## 4. Conclusions

We successfully isolated lupinifolin (**1**) and citflavanone (**2**) for the first time from *E. crista-galli*, along with lonchocarpol A (**3**), which had been discovered previously. Their chemical structure was characterized using spectroscopic methods (UV, MS, IR, ^1^H NMR, ^13^C NMR, DEPT, and HMBC). 

According to the donator acceptor map in the *in silico* test, flavanones are good antiradicals with effective electron donors, whereas standard compounds are effective electron acceptors. Flavanones are more effective electron acceptors in methanol than in the gas phase. Under the solvent effect, the three flavanones in methanol are less efficient electron donors but are better electron acceptors than in the gas phase. Depending on the surrounding chemical environment, flavanones may transport electrons via one of two possible processes: oxidation or reduction. Based on frontier molecular orbital analysis, lupinifolin (**1**) has better antiradical activity compared to the other two flavanones because this compound had the highest HOMO energy and the smallest energy gap.

Three flavanone compounds that were isolated from *E. crista-galli* twigs were evaluated for their antioxidant activity against DPPH radicals. With an IC_50_ value of 128.64 ppm, lupinifolin (**1**) demonstrated the highest antioxidant activity compared to citflavanone (**2**) and lonchocarpol A (**3**). The *in silico* studies showed similar trends to the in vitro assays using the DPPH method. 

## Figures and Tables

**Table 1:** The global descriptive parameters (in eV) of **1**–**3** and standard compounds at the B3LYP/6-31+G (2d,2p) level of theory.

Compound	*I*	*A*	*η*	*s*	*χ*	*μ*	*ω*
Lupinifolin (**1**)	7.15	0.07	3.53	104.62	3.61	−3.61	0.24
Citflavanone (**2**)	7.24	−0.00	3.62	102.14	3.61	−3.61	0.24
Lonchocarpol A (**3**)	7.24	−0.10	3.67	100.70	3.57	−3.57	0.23
Quercetin (standard)	8.03	2.99	2.51	147.01	5.51	−5.51	0.55
Ascorbic acid (standard)	9.74	3.06	3.34	110.83	6.40	−6.40	0.75

**Table 2:** Electro-donating power (*ω*^−^), electro-accepting power (*ω^+^*), and indexes (*Rd* and *Ra*) of **1–3** and standard compounds at the B3LYP/6-31+G(2d,2p) level of theory (in eV).

Compound	*ω* ^−^	ω*^+^*	*Rd*	*Ra*
Lupinifolin (**1**)	4.09	0.48	1.18	0.14
Citflavanone (**2**)	4.06	0.45	1.17	0.13
Lonchocarpol A (**3**)	3.97	0.40	1.14	0.12
Quercetin (standard)	9.10	3.59	2.62	1.05
Ascorbic acid (standard)	9.75	3.35	2.81	0.98

**Table 3:** The values of *I* and *A* for flavanones **1**–**3** in the presence of methanol solvent at the B3LYP/6-31+G (2d,2p) level of theory (in eV).

Compound	*I* (gas)	*A* (gas)	*I* (MeOH)	*A* (MeOH)
Lupinifolin (**1**)	7.15	0.07	5.77	1.71
Citflavanone (**2**)	7.24	−0.01	5.76	1.74
Lonchocarpol A (**3**)	7.24	−0.10	5.98	1.65

**Table 4:** Antioxidant activities of extracts and **1–3** of *E. crista-galli.*

Sample	IC_50_ (ppm)
*n*-hexane extract	536.47
Ethyl acetate extract	64.41
Lupinifolin (**1**)	128.64
Citflavanone (**2**)	548.72
Lonchocarpol A (**3**)	441.49
Quercetin (standard)	8.14
Ascorbic acid (standard)	4.53

## Data Availability

Data can be found in this article and the Supplementary Materials.

## Supplementary Materials

The following are available online at https://www.mdpi.com/article/10.3390/molecules27186018/s1, Table S1: The XYZ coordinates of all atoms of all structures optimized by the DFT method.

## Sample Availability

Samples of the compounds **1–3** are available from the authors.
