# Supplementary material for: Flavanones from Erythrina crista-galli Twigs and Their Antioxidant Properties Determined through In Silico and In Vitro Studies
Source: Molecules. 2022 Sep 15;27(18):6018. doi: 10.3390/molecules27186018 (PMC9501950; doi:10.3390/molecules27186018)
Supplement: Supplementary file 1 [file molecules-27-06018-s001.zip › molecules-1869169-supplementary.pdf]

molecules

MDPI

Supplementary Material for Article:

Flavanones from *Erythrina crista-galli* Twigs and Their Antioxidant Properties Determined through *In Silico* and *In Vitro* Studies

Vanny Deviani [1], Ari Hardianto [1], Kindi Farabi [1] and Tati Herlina [1,*]

Table S1. The XYZ coordinates of all atoms of all structures optimized by the DFT method.

Lupinifolin (1)

O	-3.43080	0.99250	-0.79000	C	1.78400	3.40250	-0.07780
O	1.01680	-0.35870	-0.30260	H	1.76130	-0.99660	1.50430
O	-2.49970	-3.48280	0.65170	H	2.42170	-3.26640	0.67410
O	0.07890	-4.19700	0.74680	H	1.94790	-2.79470	-0.96140
O	7.21410	0.65540	-0.77740	H	0.22800	1.62000	-1.45580
C	-1.19740	0.32230	-0.53110	H	-1.44530	2.11990	-1.64280
C	-0.29530	-0.70040	-0.20860	H	-6.38470	-0.62060	-0.39040
C	-4.80890	0.95580	-0.30170	H	-4.85650	-2.50260	-0.14200
C	-2.55690	0.01640	-0.44120	H	-5.16880	2.83210	-1.31900
C	1.96720	-1.15700	0.43780	H	-5.54710	1.39660	-2.29240
C	-0.70300	-2.00370	0.18190	H	-6.63530	1.89360	-0.98060
C	-3.03030	-1.26110	-0.07020	H	-4.20920	0.97580	1.79060
C	1.74740	-2.62590	0.10350	H	-4.45500	2.59030	1.08570
C	-2.09540	-2.25140	0.26320	H	-5.84990	1.57190	1.50100
C	0.31340	-3.05360	0.39660	H	-1.51170	2.72530	0.84680
C	-0.72250	1.70700	-0.93230	H	3.90540	-0.39040	2.18580
C	-5.31670	-0.46440	-0.28990	H	3.12830	-0.76240	-2.01730
C	-4.46610	-1.48860	-0.14290	H	-3.46020	-3.49840	0.72280
C	3.35070	-0.64680	0.12340	H	6.20140	0.37050	1.69760
C	-5.58870	1.82420	-1.28850	H	5.42930	0.01550	-2.50720
C	-4.82900	1.56310	1.10940	H	-0.61210	4.23250	2.31270
C	-0.60640	2.64490	0.24770	H	1.12370	3.99460	2.58540
C	4.23100	-0.31380	1.15350	H	0.57540	5.31840	1.56830
C	3.79830	-0.52400	-1.19910	H	1.84450	2.70910	-0.91540
C	0.45140	3.37670	0.62680	H	1.99860	4.41050	-0.45390
C	5.52980	0.11830	0.88240	H	2.59140	3.15280	0.62050
C	5.08520	-0.08500	-1.48500	H	7.72110	0.84060	0.02050
C	5.95810	0.23370	-0.44020				
C	0.37080	4.27490	1.83810				

Citflavanone (2)

O	0.62230	-0.04040	-0.05620
O	-4.05300	-0.69820	-0.32860
O	-2.39060	3.70810	0.01190
O	0.27540	4.01940	0.33070
O	6.56380	-2.08510	0.33040
C	1.73470	0.81680	-0.40390
C	-3.94530	-2.11590	0.03370
C	-0.61710	0.51430	-0.07210
C	-1.67590	-0.39930	-0.17120
C	1.66470	2.08580	0.43590
C	-0.82090	1.91520	0.01500
C	-2.98420	0.11420	-0.18850
C	0.33180	2.80490	0.25050
C	-2.56900	-2.64530	-0.29130
C	-1.50540	-1.83290	-0.34680
C	3.00470	0.02340	-0.22970
C	-2.16340	2.37640	-0.04920
C	-3.22850	1.48550	-0.14850
C	-4.23930	-2.23270	1.53680
C	-5.02650	-2.80650	-0.79690
C	3.96710	0.00230	-1.23980
C	3.26620	-0.68040	0.95380
C	5.16870	-0.68800	-1.07750
C	4.45360	-1.38170	1.12500
C	5.41250	-1.38260	0.10760
H	1.61970	1.08090	-1.46350
H	2.46850	2.77520	0.17310
H	1.77760	1.83140	1.49670
H	-2.47450	-3.71470	-0.44400
H	-0.51210	-2.21490	-0.54450
H	-4.25190	1.84170	-0.18680
H	-3.49830	-1.67810	2.11690
H	-5.23430	-1.83900	1.76260
H	-4.19780	-3.28050	1.84700
H	-5.06070	-3.87190	-0.55430
H	-6.00540	-2.37080	-0.58320
H	-4.81960	-2.69820	-1.86360
H	3.78350	0.52980	-2.17030
H	2.52600	-0.69240	1.74550
H	5.90640	-0.69190	-1.87420
H	4.65330	-1.93180	2.03650
H	-3.34160	3.86530	-0.02180
H	7.14480	-2.01110	-0.43440

Lonchocarpol A (3)

O	-1.31030	-0.26860	-0.24200
O	2.35270	-3.24890	0.57440
O	3.04760	1.22380	-1.04660
O	-0.13930	-3.90000	1.22480
O	-7.58380	0.49670	-0.19030
C	-2.22310	-1.38680	-0.28650
C	-1.89190	-2.33620	0.85620
C	0.01980	-0.55830	-0.21770
C	0.50450	-1.81490	0.22810
C	0.86590	0.47290	-0.63570
C	-0.44010	-2.80030	0.79380
C	2.78970	-1.03320	-0.25310
C	1.90720	-2.03080	0.18180
C	2.23910	0.21000	-0.62540
C	-3.63100	-0.84780	-0.25880
C	0.33520	1.82130	-1.08610
C	4.28840	-1.25560	-0.39600
C	-4.56720	-1.26490	-1.20970
C	-4.04530	0.04960	0.73090
C	0.36090	2.85520	0.01700
C	5.12980	-0.30790	0.43450
C	-5.88510	-0.81590	-1.17660
C	-5.35700	0.51440	0.77110
C	-0.63730	3.64430	0.43950
C	-6.28200	0.07810	-0.18190

C	6.22320	0.37850	0.05640
C	-0.41170	4.64070	1.55190
C	-2.03560	3.64890	-0.12450
C	6.94520	1.26960	1.03750
C	6.84850	0.32380	-1.31540
H	-2.05840	-1.90350	-1.24160
H	-2.53530	-3.21730	0.83910
H	-2.05000	-1.82670	1.81440
H	0.96100	2.17620	-1.91190
H	-0.67220	1.68980	-1.47750
H	4.56060	-2.27970	-0.11130
H	4.56080	-1.18820	-1.45420
H	-4.26510	-1.95310	-1.99260
H	-3.33510	0.40170	1.47000
H	1.32790	2.96430	0.50400
H	4.79160	-0.19180	1.46250
H	3.31460	-3.26750	0.53550
H	3.95640	1.06260	-0.74850
H	-6.60820	-1.14180	-1.91410
H	-5.66230	1.21540	1.54250
H	0.61540	4.61160	1.92240
H	-0.62410	5.66280	1.21410
H	-1.08550	4.44640	2.39590
H	-2.20530	2.87060	-0.86740
H	-2.77230	3.51430	0.67640
H	-2.25810	4.61730	-0.58990
H	6.45800	1.28100	2.01430
H	7.98020	0.93470	1.17370
H	6.99750	2.29940	0.66570
H	6.28190	-0.26910	-2.03320
H	6.96850	1.33320	-1.72470
H	7.85540	-0.10500	-1.25050
H	-7.73310	1.10980	0.53750

Quercetin

O	0.29390	0.72810	-0.18150
O	-0.35580	-2.81550	0.35850
O	4.56730	-1.45430	0.22800
O	2.33730	-2.76600	0.34660
O	4.25550	3.21510	-0.34740
O	-4.45510	2.11750	0.96830
O	-5.96230	0.29650	-0.29820
C	2.40620	-0.41930	0.02940
C	1.65820	0.76330	-0.13480
C	-0.38270	-0.44720	-0.06220
C	-1.83880	-0.26780	-0.12960
C	0.27060	-1.63360	0.14910
C	1.72660	-1.69630	0.18200
C	3.84920	-0.33610	0.06680
C	2.25830	1.99850	-0.26590
C	3.70890	2.09340	-0.23150
C	-2.42630	0.86450	0.46590
C	4.47340	0.88090	-0.06170
C	-2.66640	-1.18820	-0.79310
C	-3.79650	1.05270	0.41150
C	-4.04500	-0.99390	-0.84610
C	-4.62140	0.11900	-0.24260
H	1.67870	2.90220	-0.39800
H	-1.80230	1.59150	0.97370
H	5.55270	0.95100	-0.03640
H	-2.23510	-2.02970	-1.32200
H	-4.68660	-1.69170	-1.36950
H	-1.30290	-2.66920	0.48350
H	3.92850	-2.20880	0.30920
H	-3.83480	2.72490	1.38540
H	-6.19020	1.11860	0.15700

Ascorbic acid

O	-0.68290	-1.38130	-0.59330
O	2.55170	-0.03560	-1.27750
O	-0.05380	2.15370	-0.52690
O	3.13190	-0.28150	1.43310
O	-2.76880	1.26250	0.69160
O	-2.73400	-1.62560	0.38190
C	0.17210	-0.26880	-0.93060
C	1.57610	-0.54510	-0.36440
C	-0.54860	1.01050	-0.42430
C	1.78420	0.04980	1.01850
C	-1.74630	0.53200	0.10410
C	-1.83100	-0.87320	0.01070
H	0.26840	-0.22780	-2.02050
H	1.68390	-1.63680	-0.29660
H	1.65340	1.13330	0.96800
H	1.05810	-0.37840	1.71720
H	3.37960	0.00560	-0.78360
H	3.39050	0.33850	2.11980
H	-3.42570	0.59700	0.94060
